# Interprofessional health education teacher training at the University of Chile

**DOI:** 10.3352/jeehp.2021.18.30

**Published:** 2021-11-15

**Authors:** Mónica Espinoza Barrios, Sandra Oyarzo Torres

**Affiliations:** Department of Education in Health Sciences and Undergraduate Management, Faculty of Medicine, University of Chile, Santiago, Chile; Hallym University, Korea

**Keywords:** Educational personnel, Health education, Longitudinal studies, Role playing, Teacher training

## Abstract

The first interprofessional course that included students in the 8 undergraduate health programs at the Faculty of Medicine of the University of Chile was implemented in 2015. For the 700 students, 35 teachers were trained as facilitators. The use of several strategies to train facilitators in interprofessional health education, such as working in small groups, role-playing, case analysis, personal development workshops with experts’ participation, teamwork skills, feedback, videos, and reading articles, proved to be helpful. Facilitators highlighted the use of syllabi as a fundamental tool for teaching and coordination. This guide describes the experience of interprofessional health education teacher training from 2015 to 2019, highlighting the following lessons learned: the importance of support from university authorities, raising faculty awareness about interprofessional health education and collaborative practice, creating a teachers’ coordination team including representatives from all health programs, and ongoing monitoring and feedback from participants.

## Introduction

This guide intends to disseminate the strategies utilized to train teachers as interprofessional health education (IPE) facilitators in a purpose-built IPE course from 2015–2019 at the Faculty of Medicine of the University of Chile.

The available evidence on this topic shows several challenges, especially concerning the training of a team, mainly due to the central role that facilitators play in implementing and modeling IPE. The most relevant challenge for IPE teachers’ training lies in the great diversity of professionals involved in the courses—with different disciplines, professions, experiences, levels of teamwork skills—in addition to the lack of experience and knowledge of IPE and the absence of national policies to promote IPE. We have found similar difficulties to those reported in the international literature concerning the institutional and personal barriers that prevent facilitators from engaging as leaders [1].

## Implementing IPE at the Faculty of Medicine of the University of Chile

**Fig. 1.**
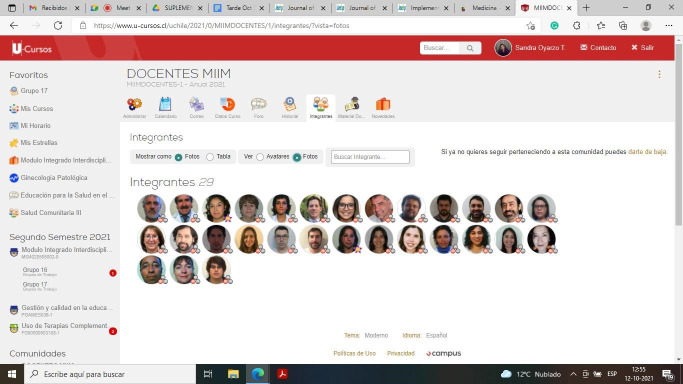
Team of interprofessional education facilitators at the Faculty of Medicine of the University of Chile.

In 2009, a team of facilitators responsible for designing and implementing IPE courses was formed, including professionals from the Science, Health, and Education Departments and Units. They met weekly to reflect upon, discuss, elaborate, and implement the proposal. Their work consisted of defining the competencies, achievement indicators and levels, the course development methodology, assessment instruments, preparation of the necessary documents for the course, qualifications required for academics to participate in the course, resources required for the course, and logistical aspects of its implementation. The pilot course included students from 2 undergraduate programs in 2010. In 2015, the course was implemented for the first time for the Faculty’s 8 undergraduate programs (Supplements 1, 2), with participation by 700 students and 35 teachers in charge (Fig. 1). A teaching community, an interprofessional coordinating team, a teaching secretariat supported by the facilitator development unit, and a psychologist from the human resources unit also participated in the project. The second IPE experience occurred in 2016 and included the 8 undergraduate programs. Five hundred students, 30 teachers in charge, 50 student sections, and a multi-professional coordinating team participated in this phase [2]. Information about the IPE program can be accessed through the Faculty of Medicine of the University of Chile (http://www.medicina.uchile.cl/; http://formacioncomun.med.uchile.cl).

## Lessons learned

The lessons learned below were derived from the teachers’ evaluations of the IPE process.

### Raise awareness of IPE and collaborative practice before participation in the IPE teachers’ training program.

Facilitators need to be fully aware of the meaning and rationale of IPE. They must complete the IPE facilitator training program, focusing on the skills they need to develop for this purpose [3].

### Ensure the training of an IPE leader teaching coordination team with adequate competencies and motivation comprising individuals affiliated with the health programs mentioned above.

Implementing an IPE teacher training program requires forming a leadership coordination team made up of teachers affiliated with varied health and science programs, with experience in teamwork skills, collaborative practice, interprofessional leadership, and effective communication, as well as (ideally) with a background in health sciences education. Successfully completing this step requires collaborative leadership, shared decisions, and appreciation of each health profession’s different perspectives [4].

### Know and implement the best strategies for teacher training in IPE based on the best evidence available.

Initially, the training was conducted in 1 or 2 sessions before the course started and in 3 course sessions to monitor the process. In these sessions, teachers had the opportunity to share their positive and negative experiences during the course, and a psychologist provided them with strategies for teamwork and conflict resolution in their sessions. This empowerment model was used for 3 years. However, academics reported that they needed more tools to participate in these courses. In our experience, the most successful teacher training model consisted of implementing a more personalized design that reinforced peer work and prompted teachers to participate in all sessions in the way that students would participate [5].

### Accompany teaching teams during the implementation of the course, both in-person and virtually.

From the beginning, teachers were accompanied in the course’s implementation individually and in groups; in the first versions, this accompaniment was performed by a psychologist who specialized in teamwork to reinforce teamwork competencies. As another form of support, a teaching community was created on the institutional virtual platform, with access to discussion forums, email communication, documents, and videos that allowed support and response to the needs that were expressed [6].

### Seek expert advice on IPE to strengthen the program.

Expert advice on IPE is recommended in the program design, implementation and evaluation phases to decide on the best spaces within the curriculum, activities, teaching strategies, and assessment tools to measure IPE skills such as effective communication, role definition, conflict resolution, and people-centered care [7].

### Create a syllabus.

It was necessary to create a syllabus consisting of a guide that described the work to be done by the teacher in each session and established standard guidelines, a statement of the expected learning achievements for students in each session, activities to be carried out (e.g., reading articles, viewing of videos, case analysis, and completion of the work guide), the time allocated to each activity, session evaluation, and recommendations for alternative activities [2].

### Seek out support from faculty leadership and recognition of the teaching profession.

One of the central elements in developing an IPE program is the endorsement of the importance of IPE training in health professionals by authorities such as the dean, the undergraduate director, and heads of departments and health programs. This support translates into a commitment to developing IPE in the educational institution, providing financial resources, disseminating IPE through the institution’s communication networks, and developing future IPE projects [8].

### Promote spaces to disseminate knowledge and scientific evidence that supports IPE and its importance to develop undergraduate health programs and personal care.

Holding IPE conferences makes it possible to share evidence-based practices and learning for teachers who participate in these initiatives. Joining national and international networks on IPE and collaborative practice enables exchanges of experiences, ideas, visions on IPE, collaborative actions, and good practices among different IPE networks, thereby facilitating teachers’ mobility [9].

### Regularly monitor course progress and outcomes, the key to teamwork in IPE.

Regular course evaluations to achieve success in the implementation and development of the IPE courses were required, as well as teams of teachers and students who continuously evaluated the course development through regular coordination meetings, interviews, surveys, peer evaluation, team evaluations, and ongoing feedback [10].

## Conclusion

The lessons learned for training teachers as facilitators in an IPE course included, firstly, the need to obtain support from the university authorities, who were an essential element to initiate the process of change and implementation of the primary resources. In addition, the awareness of the IPE teaching community and collaborative practice among academics contributed to the advancement of this experience, transforming it into a paradigm shift that sought to move towards shared decision-making. The appraisal of each of the health team members was regarded as central to the process, and thus essential for valuing IPE as a strategy for better health care, as demonstrated in research conducted elsewhere. This experience’s success was due to several factors, particularly the introduction of an interprofessional teaching coordination team composed of professionals from all health and basic sciences programs. Collaborative leadership, shared decisions, and valuing different professional views served as good models of IPE for students. Finally, for the course’s implementation and development to succeed, it was vital to carry out ongoing monitoring and feedback of the IPE experience.

## Supplementary materials

Supplementary files are available from Harvard Dataverse: https://doi.org/10.7910/DVN/YDIZXQ

Supplement 1. Interprofessional education course program at the Faculty of Medicine of the University of Chile in 2021 (in Spanish).

Supplement 2. Interprofessional education facilitator’s syllabus module I at the Faculty of Medicine of the University of Chile in 2021 (in Spanish).

Supplement 3. Audio recording of the abstract.
