# Supplementary material for: Interprofessional health education teacher training at the University of Chile
Source: J Educ Eval Health Prof. 2021 Nov 15;18:30. doi: 10.3352/jeehp.2021.18.30 (PMC8666264; doi:10.3352/jeehp.2021.18.30)
Supplement: Supplementary file 1 — Supplement 1. Interprofessional education course program at the Faculty of Medicine of the University of Chile in 2021 (in Spanish). [file jeehp-18-30-suppl1.pdf]

PROGRAMA DE CURSO 2021

Esta versión del curso se considera excepcional, debido a la emergencia sanitaria por COVID-19. Las metodologías, calendarios y evaluaciones pueden sufrir modificaciones en el transcurso del semestre, con la finalidad de dar cumplimientos satisfactorios a los resultados de aprendizaje declarados y el propósito formativo comprometido. Los eventuales cambios se llevarán a cabo según la contingencia, serán validados por la Dirección de Pregrado y se informarán de manera oportuna a sus participantes, a través de los canales formales institucionales.

Unidad académica: Dirección de Pregrado

Nombre del curso: MÓDULO INTEGRADO INTERDISCIPLINARIO MULTIPROFESIONAL I 2021

Código: MI02020505001-1

Carrera: Enfermería, Fonoaudiología, Kinesiología, Medicina, Nutrición, Obstetricia, Tecnología Médica, Terapia Ocupacional

Tipo de curso: Obligatorio

Línea formativa: Tercer Nivel

Semestre: Primer semestre

Año: 2021

Requisitos: Sin requisitos

N° de estudiantes: 700

N°	Nombre Profesor	Depto./ Escuela a la cual pertenece
1	Prof. Fernando Valiente Echeverría	Programa de Virología. ICBM
2	Prof. Gonzalo Barriga Pinto	Programa de Virología ICBM
3	Prof. Germán Ebensperger Darrouy	Programa de Fisiopatología ICBM
4	Prof. Américo López Rivera	Programa de Fisiología. ICBM
5	Prof. Leandro Carreño Marquez	Programa Inmunología. ICBM
6	Prof. Emilio Herrera Videla	Programa de Fisiopatología ICBM
7	Prof. Aldo Gaggero Brillouet	Programa de Virología. ICBM
8	Prof. Jonás Chnaiderman	Programa de Virología ICBM
9	Prof. Nicole Herrera Toro	Departamento de Tecnología Médica
10	Prof. Jimena Valero Saavedra	Departamento de Enfermería
11	Prof. Wladimir Torres Reyes	Departamento de Enfermería

12	Prof. Jessica García Gutiérrez	Departamento de Enfermería
13	Prof.Paulina Larrondo	DIGEN
14	Prof. Carmen Paz Díaz Camazón	Departamento de Escuela Terapia Ocupacional
15	Prof. Lissette Duarte Silva	Departamento de Nutrición
16	Prof. Natalia Gómez San Carlos	Departamento de Nutrición
17	Prof. Matías Henríquez Ortiz	Depto. Promoción de la Salud de la mujer y recién nacido
18	Prof. Pamela Soto Herrera	Depto. Promoción de la Salud de la mujer y recién nacido
19	Prof. Mónica Espinoza Barrios	Departamento de Educación en Ciencias de la Salud. DECSA
20	Prof. Sandra Oyarzo Torres	Departamento de Educación en Ciencias de la Salud. DECSA
21	Prof. Tomás Hernández González	Departamento de Kinesiología
22	Prof. Paola Gaete	Departamento de salud de la mujer y recién nacido

23	Prof. Cristobal Catalan	Departamento de Terapia Ocupacional
24	Prof. Claudia Goya	Departamento Tecnología Médica
25	Prof. Hernán Aguilera	Dirección de Extensión
26	Prof. Vivian Luchsinger	Programa de Virología ICBM

En el año 2010 la Organización Mundial de la Salud (OMS) en el marco de acción de la educación interprofesional y cuidado colaborativo, destacó la importancia de la educación interprofesional para promover modelos sustentables basados en trabajo en equipo interdisciplinario para los cuidados de las personas en salud.

La educación interprofesional (EIP) apunta a mejorar la colaboración entre distintos tipos de profesionales de la salud y de la atención social para facilitar la colaboración y el cuidado de las personas. Según la definición de CAIPE (Centre for the Advancement of Interprofessional Education https://www.caipe.org) ésta se logra cuando: "estudiantes de dos o más profesiones aprenden con el otro, del otro y acerca del otro, para colaborar en la práctica profesional".

La Educación Interprofesional se ha introducido ampliamente como un tema importante a abordar desde diferentes perspectivas en la formación de profesionales de la salud, con la finalidad de poder brindar una atención integral y centrada en las personas, mejorando la calidad de vida de las comunidades. La incorporación de los modelos curriculares interprofesionales en los planes de formación de los estudiantes de la salud, ha sido promovido como un importante avance para dar respuesta a las actuales demandas de los sistemas de salud y la comunidad.

La Universidad de Chile, en su permanente compromiso con los lineamientos de la OMS, respondió a la necesidad de implementar el aprendizaje colaborativo entre las ocho carreras de la salud en la Facultad de Medicina, mediante la creación de los Módulos Integrados Interdisciplinarios Multiprofesionales (MIIM 1 y MIIM 2). Estos cursos son obligatorios para todos los estudiantes de salud durante su tercer y cuarto año de estudios específicos de disciplina. Durante estos cursos estudiantes de Obstetricia, Enfermería, Terapia Ocupacional, Kinesiología, Tecnología Médica, Fonoaudiología, Medicina y Nutrición deben trabajar juntos para lograr los objetivos del curso basados en el trabajo en equipo. Los módulos MIIM 1 y MIIM 2 en particular, representan un esfuerzo intercurricular que beneficia a los estudiantes y docentes de la Universidad de Chile involucrados en las profesiones de la salud.

PROPÓSITO FORMATIVO

Este curso pretende favorecer el desarrollo en el estudiante de habilidades para el trabajo en equipo interprofesional y la práctica colaborativa, abordando situaciones que faciliten la aplicación de estos aprendizajes e identificando los roles y contribuciones de cada una de las profesiones, en el marco de la seguridad de las personas, familias y comunidades en la atención de salud.

Es la primera instancia de educación interprofesional que le permite al estudiante la integración de saberes previos y reconocerse como parte de un equipo de salud, en un ambiente de trabajo respetuoso que promueva una atención de salud de calidad, integral y centrada en las personas

Se relaciona curricularmente con MIIM II del Octavo semestre

COMPETENCIAS DEL CURSO
Las Competencias mencionadas a continuación fueron seleccionadas por las Escuelas desde los Perfiles de Egreso de las respectivas carreras:

<u>Tecnología Médica:</u>

Dominio Genérico Transversal

C2. Ser un profesional crítico y reflexivo en las decisiones, acciones y procedimientos que realiza para contribuir eficazmente en los distintos ámbitos o dominios de desempeño del Tecnólogo(a) Médico(a).
Subcompetencias 2.1
Actuando analítica y reflexivamente, con una visión de la complejidad de los procesos y de su contexto.
Sub Competencias 2.2
Argumentando por medio de la lógica, sus decisiones en su quehacer profesional.

C3. Utilizar herramientas de aproximación a las personas de acuerdo a sus características individuales, a su contexto grupal y social para interactuar de manera pertinente a la situación y para obtener la información necesaria que permita decidir las acciones a desarrollar en su ámbito profesional.
Subcompetencia 3.1
Utilizando eficazmente la comunicación verbal, corporal y escrita para facilitar y optimizar la comprensión del mensaje.

<u>Terapia Ocupacional:</u>

Dominio Genérico

C.6 Establecer relaciones profesionales e interpersonales asertivas, empáticas, y honestas con las personas y grupos, en forma creativa y con liderazgo en diferentes contextos y ambientes.
Subcompetencia 6.1 Comprendiendo los distintos elementos involucrados en el trabajo profesional y las relaciones interpersonales en la intervención terapéutica con personas y grupos.

Subcompetencia 6.2 Desarrollando creativamente habilidades interpersonales para desempeñar el rol profesional.
Subcompetencia 6.3 Promoviendo un ambiente de trabajo en donde la convivencia en la diferencia es considerada fuente de riqueza de las relaciones interpersonales.
Subcompetencia 6.4 Colaborando activamente y priorizando los intereses del colectivo antes de los propios, para el logro de una tarea común.

<u>Medicina:</u>.

Dominio ético social

C2. Integra equipos de trabajo ejerciendo liderazgo participativo y colaborativo, con flexibilidad y adaptabilidad a los cambios, con una actitud permanente de servicio y compromiso en los diversos ámbitos en los que se desempeña.

Subcompetencia 2.1 Contribuye al crecimiento y superación de las debilidades, y potencia las fortalezas tanto personales, como del equipo de salud y la comunidad, que influyen directa o indirectamente en la situación de salud.
Subcompetencia 2.2 Contribuye activamente con flexibilidad y adaptabilidad para resolver situaciones de conflicto e incertidumbre generadas en el equipo de salud, buscando la solución en forma participativa y colaborativa.

<u>Enfermería</u>

Dominio Genérico

EG 3: Proponer juicios reflexivos mediante el análisis de conceptos, procesos y resultados de las propias acciones y las de otros, basado en criterios teóricos, metodológicos e ideológicos y establecer acciones de ser necesario, en sus distintas áreas formativas.
EG 3.2: Reflexionando frente a diversas situaciones, pensamientos y posturas que se le presentan durante el proceso formativo.

<u>Kinesiología</u>

Dominio Genérico Transversales

KGT3. Establece relaciones interpersonales efectivas.
[illegible]. Se comunica de manera efectiva, aplicando principios de asertividad y empatía en sus relaciones cotidianas.
[illegible]. Se desempeña de manera proactiva y efectiva en equipo.
[illegible]. Aplica principios éticos en su actuar profesional y cotidiano.

<u>Fonoaudiología</u>

Dominio responsabilidad social:

C2: Ejerce su labor profesional, valorando la diversidad de las personas y respetando el entorno.
SC2.1: Demostrando una actitud inclusiva y tolerante en su relación con grupos diversos.

Dominio genérico transversal:
C1: Utiliza herramientas de comunicación que le permiten interactuar adecuadamente en distintos contextos
SC1.1: Utilizando eficazmente la comunicación verbal, corporal y escrita para facilitar y optimizar la comprensión del mensaje.
C2: Integra equipos de trabajo, con el fin de desarrollar acciones que contribuyan a la comprensión y solución de situaciones fonoaudiológicas.
SC2.1: Actuando comprometidamente en los equipos de trabajo.
SC2.2: Fortaleciendo el entendimiento y construyendo acuerdos entre los diferentes actores
SC3.1: valorando el protagonismo y la responsabilidad de las personas y de los grupos sociales en la detección de necesidades y la construcción colectiva de respuestas.

<u>Obstetricia</u>

Dominio Genérico – Transversal:

C1 Establecer una comunicación efectiva que evidencie una relación empática, asertiva y honesta con las personas, considerando su nivel de educación, etnia, cultura, y religión, en las diversas situaciones que debe enfrentar en su formación profesional.
Subcompetencia 1.1 Incorporando elementos del desarrollo personal que le permitan relacionarse asertivamente con los diferentes integrantes de la comunidad.
C4 Trabajar en equipo, identificando las potencialidades y delimitando las responsabilidades propias como las del resto del grupo, priorizando los intereses del colectivo antes de los propios, para el logro de una tarea común, en los términos, plazos y condiciones fijados en los diversos contextos de su formación.
Subcompetencia 4.1 Incorporando elementos del desarrollo personal que le permitan integrarse al trabajo en equipo.
Subcompetencia 4.2 Reconociendo características personales que le permitan desarrollar liderazgo dentro de los equipos de trabajo.
Subcompetencia 4.3 Potenciando sus características personales, que le permitan convertirse en un/a líder positivo/a dentro de los equipos de salud.

<u>Nutrición</u>

Dominio genérico transversal:
Competencias DGTR.C03 Actuar comprometida y activamente de manera individual o en equipos de trabajo en la búsqueda y desarrollo de acciones que contribuyan a mejorar las situaciones que afectan la alimentación, nutrición y salud de individuos y poblaciones
Subcompetencia DGTR.C03.S01 Participando activamente en equipos de trabajo disciplinares y multidisciplinarios en el contexto
profesional
Competencias DGTR.C02 Interactuar con individuos y grupos, mediante una efectiva comunicación

verbal y no verbal, en la perspectiva de fortalecer el entendimiento y construir acuerdos entre los diferentes actores, teniendo en cuenta el contexto sociocultural y la situación a abordar, acorde con principios éticos.<br>**Subcompetencia DGTR.C02.S01** Comunicándose de manera efectiva con individuos y grupos, considerando aspectos como asertividad, empatía, respeto, entre otras, de acuerdo al contexto sociocultural.

RESULTADOS DE APRENDIZAJE DEL CURSO:

Unidad I: Introducción a la Educación Interprofesional

- Analizar el fundamento de la EIP en la atención integral de las personas y práctica colaborativa.
- Analizar los roles de los/las/les integrantes del equipo de salud y la contribución de estos en la atención de las personas.

Unidad II: Habilidades para el trabajo en equipo y práctica colaborativa

- Reflexionar sobre las habilidades fundamentales en los equipos interprofesionales que favorecen la comunicación efectiva, la resolución de conflictos y el liderazgo cooperativo.

Unidad III: Integración del trabajo interprofesional y seguridad en los cuidados de salud

- Aplicar los fundamento de la EIP y práctica colaborativa para disminuir los eventos adversos y favorecer una práctica de atención de salud interprofesional segura para las personas.

PLAN DE TRABAJO

Unidades de Aprendizaje	Logros de Aprendizaje	Acciones Asociadas
El horario de este de 15:00 a 18:00 se distribuirá en: El trabajo se llevará a cabo durante la jornada de la tarde, la cual estará dividida en dos: una parte presencial con el PEC y la segunda mitad para trabajo del		

equipo MIIM independiente. Los equipos irán alternando sus horarios de trabajo.		
**Unidad I:**<br>**Introducción a la Educación Interprofesional**	**Sesión 1:**<br>**Con el PEC presencial**<br>Presentación del Programa de curso, PEC y de los integrantes del equipo MIIM<br><br>**Sin el PEC los estudiantes trabajan independientes**<br>Analizar los conceptos generales de la Educación interprofesional<br><br>Analizar lo factores que condicionan la práctica colaborativa interprofesional<br><br>Reflexionar acerca del fundamento de la Educación Interprofesional en el cuidado de la salud de las personas.<br><br>**Sesión 2**<br>Analizar los roles del equipo de salud y la contribución de cada uno/a/e de ellos/as/es en el cuidado de las personas y comunidad<br><br>Relacionar la EIP y práctica colaborativa en el cuidado de las personas y comunidad<br><br>Analizar las características de los equipos de trabajo dentro de las organizaciones.	**Sesión 1:**<br>Cada integrante del equipo se presenta, de acuerdo con la actividad indicada por el PEC<br><br>Observa video, realiza lectura de documento y reflexiona junto a sus compañeros/as/es sobre el fundamento de la Educación Interprofesional<br>Video 1: Contextualización EIP<br>https://www.youtube.com/watch?v=NcAOl_fYP90<br>Video 2: Cómo formar a los profesionales sanitarios<br>https://drive.google.com/drive/u/1/folders/1pkNIGQqEkrh5C_iGVLxA3S2mbOTLRcpE<br><br>Trabaja junto a sus compañeros/as/es en el desarrollo de la pauta guía la cual será revisada en la siguiente sesión.<br><br>**Sesión 2**<br>Analiza los perfiles de egreso de las diferentes profesiones que constituyen el equipo de salud.<br><br>Observa los videos extrayendo los elementos relacionados con la EIP y práctica colaborativa.<br><br>Trabaja junto a sus compañeros/as/es en el desarrollo de la pauta guía la cual subirán a U- cursos, una vez que esté completa.

	**Sesión 3**<br><br>Analizar los roles del equipo de salud y la contribución de cada uno/a/e de ellos/as/es en el cuidado de las personas y comunidad<br><br>Comprender las características de los equipos de trabajo dentro de las organizaciones.<br><br>Analizar las etapas de la constitución de un equipo de trabajo<br><br>**Sesión 4**<br>Analizar los elementos favorecedores y obstaculizadores del trabajo interprofesional y práctica colaborativa<br><br>**Sesión 5**<br>Realizar análisis crítico Integrando los elementos aprendidos sobre EIP y práctica colaborativa en el trabajo realizado con sus compañeros en esta unidad	**Sesión 3**<br><br>Trabaja junto a sus compañeros/as/es confeccionando mensajes clave relacionados al Trabajo en equipo y roles de los integrantes del equipo de salud, incluyendo lo aprendido en ambas sesiones para difundir a través de la plataforma de Instagram.<br><br>**Sesión 4**<br>Actividad integradora de la Unidad I, Trabaja junto a sus compañeros en el desarrollo de un caso integrador de EIP y práctica colaborativa.<br>**(Tiempo protegido para trabajo con sus compañeros)**<br><br>**Sesión 5**<br>Realiza junto a sus compañeros informe final de la Unidad 1 a través de presentación al PEC, y sube material a ícono tareas de u cursos, según fecha indicada en pauta guía.

**Unidad II:**<br>**Habilidades para el trabajo en equipo interprofesional y práctica colaborativa**	**Sesión 6:**<br><br>Analizar críticamente las competencias fundamentales para el trabajo en equipo interprofesional y práctica colaborativa<br><br>Identificar los factores que contribuyen a una colaboración exitosa en los equipos de trabajo<br><br>**Sesión 7:**<br><br>Analizar la comunicación efectiva en el trabajo en equipo interprofesional y su repercusión en los cuidados de las personas<br><br>Identificar los elementos que facilitan y obstaculizan la comunicación verbal y no verbal en el contexto del trabajo en equipo de salud.<br><br>**Sesión 8:**<br>Analizar las causas del conflicto y proponer estrategias de resolución del conflicto	**Sesión 6:**<br><br>Analizar críticamente junto a sus compañeros las competencias fundamentales para el trabajo en equipo interprofesional a través del análisis de un video y artículo.<br>(Resolver pauta guía)<br><br>Construir un mapa conceptual que muestre las características y los desafíos de un buen trabajo en equipo interprofesional y publicar en Instagram.<br>**(Tiempo protegido para trabajo con sus compañeros)**<br><br>**Sesión 7:**<br><br>Reflexionar junto a sus compañeros las habilidades necesarias para la comunicación efectiva dentro de los equipos de salud a través del análisis de un video y artículo.<br>**(Resolver pauta guía junto a su equipo y PEC durante la sesión sincrónica)**<br><br>**Sesión 8:**<br>Lectura del capítulo “conflicto en los equipos” disponible en páginas 60 a 70, del documento Trabajo en equipos de salud.<br>Analizar una noticia relacionada con alguna problemática a causa de la pandemia y que pudiera afectar a los equipos de salud generando conflicto

	**Sesión 9:**<br>Analizar los elementos del liderazgo colaborativo en los equipos interprofesionales<br><br>Reflexionar sobre los diferentes modelos de liderazgo que se pueden ejercer en los equipos de salud<br><br>**Sesión 10:**<br>Analizar las estrategias adecuadas para la resolución de conflictos en el marco del trabajo en equipo	Reflexionar en base a la pauta guía.<br><br>**Sesión 9:**<br>Realiza análisis crítico junto a sus compañeros sobre los elementos necesarios para el liderazgo y práctica colaborativa (lectura artículo y vídeos) en la resolución de los conflictos<br><br>Responder junto a sus compañeros la pauta guía de preguntas durante la sesión.<br><br>**Sesión 10:**<br>Realizar entrevista a profesionales de la salud acerca de sus experiencias en trabajo de equipo de salud interprofesional<br><br>Reflexionar acerca de los aprendizajes de esta unidad, habilidades fundamentales del trabajo interprofesional en salud.<br><br>Enviar un audio o video individual acerca de sus conclusiones, de los aprendizajes en esta unidad.
**Unidad III: Integración del trabajo interprofesional y seguridad en los cuidados de salud**	**Sesión 11:**<br>Analizar la estrategia de EIP y práctica colaborativa de la OMS<br><br>Identificar las causas de los eventos adversos en salud<br><br>Analizar las conductas erradas más frecuentes y el impacto en la atención de salud de las personas	**Sesión 11:**<br>Reflexionar junto a sus compañeros sobre la estrategia de EIP y práctica y su impacto en la disminución de los errores en los equipos de salud (Video y artículo)<br>(Resolver pauta guía)<br>**(Tiempo protegido para trabajo con sus compañeros)**

	**Sesión 12:**<br>Integrar lo aprendido en la sesión 1, elementos de la dinámica de los equipos, y factores que contribuyen al error y que pueden afectar la seguridad en la atención de las personas.<br><br>Proponer qué acciones pueden llevar a cabo los equipos interprofesionales para mejorar la seguridad del paciente.<br><br>**Sesión 13 y 14:**<br><br>Elaborar un video acerca del fundamento de la EIP y práctica colaborativa para disminuir los eventos adversos y lograr una práctica de atención interprofesional de salud segura para las personas en el marco de la Pandemia por Covid 19<br><br>**Sesión 15:**<br><br>Fundamentar los contenidos incluidos en el video acerca del fundamento de la EIP y práctica colaborativa para disminuir los eventos adversos y lograr una práctica de atención interprofesional de salud segura para las personas en el marco de la Pandemia por Covid 19	**Sesión 12:**<br>Realiza actividad de retroalimentación de la sesión 1 junto a sus compañeros/as y profesor/ra<br><br>**Comparte** junto a sus compañeros y PEC las acciones pueden llevar a cabo los equipos interprofesionales para mejorar la seguridad de las/los/les personas.<br><br>**Sesión 13 y 14**<br><br>Preparara un video junto a su equipo de trabajo<br>**(Tiempo protegido para trabajo con sus compañeros)**<br><br>Sube video a canal de YouTube<br><br>**Sesión 15:**<br><br>**Actividad Final de Unidad 3**<br>Comparte junto a sus compañeros y PEC los contenidos incluidos en el video acerca del fundamento de la EIP y práctica colaborativa para disminuir los eventos adversos y lograr una práctica de atención interprofesional de salud segura para las personas en el marco de la Pandemia por Covid 19

ESTRATEGIAS METODOLÓGICAS

- Aula invertida
- Lectura de artículos
- Videos
- Audios
- Trabajo en equipo
- Resolución de casos y pautas guías

PROCEDIMIENTOS EVALUATIVOS

Unidad 1 Introducción a la Educación Interprofesional (30%)

EVALUACIÓN INDIVIDUAL UNIDAD I 30%

RÚBRICA DE EVALUACIÓN EQUIPO INFORME ESCRITO UNIDAD I 70%

PAUTA DE COEVALUACIÓN FORMATIVA

Unidad 2 Habilidades para el trabajo en equipo interprofesional y práctica colaborativa (30%)

EVALUACIÓN INDIVIDUAL UNIDAD II 70%

PAUTA DE COEVALUACIÓN 30%

Unidad 3 Integración del trabajo interprofesional y seguridad en los cuidados de salud (40%)

EVALUACIÓN INDIVIDUAL UNIDAD III 20%

PAUTA DE COEVALUACIÓN 20%

RÚBRICA DE EVALUACIÓN DE MEDIOS AUDIOVISUALES UNIDAD III 60%

BIBLIOGRAFÍA Y RECURSOS

Obligatoria

Unidad I

M. Miró Bonet.Práctica colaborativa interprofesional en salud: Conceptos clave, factores y percepciones de los profesionales. Educ Med. 2016;17(Supl 1):21-24

Ministerio de Salud Argentina . Módulo 8 Trabajo en equipo en salud.salud. gov.ar

https://bancos.salud.gob.ar/sites/default/files/2018-10/0000001033cnt-modulo_8_trabajo-equipos-salud.pdf

OPS.La educación interprofesional para la salud universal.

https://www.paho.org/hq/index.php?option=com_content&view=article&id=14495:la-educacion-interprofesional-para-la-salud-universal-triptico-2018&Itemid=39594&lang=pt

CAIPE. Introducción a la educación interprofesional. 2013

https://www.educacioninterprofesional.org/sites/default/files/fulltext/2018/pub_caipe_intro_eip_es.pdf

Unidad II

Diego Ayuso Murillo, Máximo A. González Jurado. EL LIDERAZGO EN LOS ENTORNOS SANITARIOS Formas de gestión . Capítulo 1 : Liderazgo Sanitario,pág 1 - 23. 1° Ed. 2017.ISBN: 9788490520796

https://www.editdiazdesantos.com/wwwdat/pdf/9788490520796.pdf

Nancarrow et al. Ten principles of good interdisciplinary team work. Human Resources for Health 2013, 11:19

West. M, West. T. Leadership in Healthcare: A Review of the Evidence.HealthManagement, Volume 15 - Issue 2, 2015

Royal College of Physicians .Improving teams in healthcare Resource 3: Team communication.2017

Karen Deeny and Pip Hardy. DNA of Care facilitator's guide,Compassionate leadership.Pilgrim Projects Limited. 2018

Tony Smith, Sally Fowler Davis, Susan Nancarrow, Steven Ariss & Pam Enderby (2020) Towards a theoretical framework for Integrated Team Leadership (IgTL), Journal of Interprofessional Care, 34:6, 726-736, DOI: 10.1080/13561820.2019.1676209

Unidad III

World Health Organization. Patient Safety Workshop. LEARNING FROM ERROR. 2008

https://apps.who.int/iris/handle/10665/44267

Ministerio de Salud Argentina . Módulo 8 Trabajo en equipo en salud.salud. gov.ar

https://bancos.salud.gob.ar/sites/default/files/2018-10/0000001033cnt-modulo_8_trabajo-equipos-salud.pdf

OMS. Seguridad del paciente. 2019 (página español)

https://www.who.int/es/news-room/fact-sheets/detail/patient-safety

https://www.who.int/es/news-room/detail/13-09-2019-who-calls-for-urgent-action-to-reduce-patient-harm-in-healthcare

https://www.who.int/patientsafety/information_centre/documents/ps_research_brochure_es.pdf?ua=1

McKimm J. Giving effective feedback. Br J Hosp Med (Lond). 2009 Mar;70(3):158-61. doi: 10.12968/hmed.2009.70.3.40570. PMID: 19274007.

https://pubmed.ncbi.nlm.nih.gov/19274007/

GUÍA PARA UNA SESIÓN DE RETROALIMENTACIÓN. Traducido y adaptado por Dr. Pedro Herskovic y Dra. Natasha Kunakov. Curso docencia efectiva. DECSA. Fac Medicina. Universidad de Chile. 2020

Pauta Guía feedback positivo en el contexto del trabajo en equipo de salud. Sesión 3. Construida por Mónica Espinoza B y Sandra Oyarzo T. Programa MIIM 1. Unidad II. Unidad de Formación común. Dirección de Pregrado. Facultad de Medicina. Universidad de Chile. 2019.

Bibliografía complementaria:

Red Regional de Educación Interprofesional de las Américas y Organización Panamericana de la Salud. (2019). Atención centrada en el paciente en la práctica interprofesional colaborativa: elementos clave, estrategias y próximos pasos. Washington DC

https://www.educacioninterprofesional.org/sites/default/files/fulltext/2019/note_eip_pc_abril.pdf

Scott Reeves, Simon Fletcher, Hugh Barr, Ivan Birch, Sylvain Boet, Nigel Davies, Angus McFadyen, Josette Rivera Simon Kitto (2016) A BEME systematic review of the effects of interprofessional education: BEME Guide No. 39, Medical Teacher, 38:7, 656-668, DOI: 10.3109/0142159X.2016.1173663

Beunza Juan José & Icaran Eva. Manual de Educación Interprofesional Sanitaria. Elsevier. 2017.

George Dantas Azevedoa, Nildo Alves Batistab, Sylvia Helena Souza da Silva Batistab, Maria Isabel Barros Bellinic,Ana Maria Chagas Sette Câmarad, Marcelo Viana da Costae, Antonio Pithon Cyrinof, Eliana Goldfarb Cyrinof,Marina Peduzzig and Scott Reevesh *Interprofessional education in Brazil: Building synergic networks of educational and healthcare processes.* Journal of Interprofessional Care 2016, vol. 30, no. 2, 135–137

Costa Marcelo Viana da. *A educação interprofissional no contexto brasileiro: algumas reflexões.* Interface (Botucatu). 2016; 20(56): 197-198.

Silvana Castillo-Parra, Sandra Oyarzo Torres, Mónica Espinoza Barrios, Ana María Rojas-Serey, Juan Diego Maya, Valeria Sabaj Diez, Verónica Aliaga Castillo, Manuel Castillo Niño, Luis Romero Romero, Jennifer Foster & Gustavo Hawes Barrios (2017) The implementation of multiple interprofessional integrated modules by health sciences faculty in Chile, Journal of Interprofessional Care, 31:6, 777-780, DOI: 10.1080/13561820.2017.1345872

Jill Thistlethwaite, Kathy Dallest, Monica Moran, Roger Dunston, Chris Roberts, Diann Eley, Fiona Bogossian, Dawn Forman, Lesley Bainbridge, Donna Drynan & Sue Fyfe (2016) Introducing the individual Teamwork Observation and Feedback Tool (iTOFT): Development and description of a new interprofessional teamwork measure, Journal of Interprofessional Care, 30:4, 526-528, DOI: 10.3109/13561820.2016.1169262

Burgess, A., van Diggele, C., Roberts, C. *et al.* Feedback in the clinical setting. *BMC Med Educ* 20, 460 (2020). https://doi.org/10.1186/s12909-020-02280-5

Juan José Beunza, Eva Icaran Francisco. Manual de Educación Interprofesional Sanitaria. Primera Edición. ISBN 978-84-9113-296-7. Elsevier. Barcelona. España. 2018

McKimm J. Giving effective feedback. Br J Hosp Med (Lond). 2009 Mar;70(3):158-61. doi: 10.12968/hmed.2009.70.3.40570. PMID: 19274007.

Red Regional de Educación Interprofesional de las Américas.
https://www.educacioninterprofesional.org/es
https://www.educacioninterprofesional.org/es/recursos/publicaciones

Centre for the Advancement of Interprofessional Education
https://www.caipe.org/

Global Confederation for Interprofessional education & collaborative practice
https://interprofessional.global/

REQUISITOS DE APROBACIÓN

Artículo 24: *El rendimiento académico de los estudiantes será calificado en la escala de notas de 1,0 a 7. La nota mínima de aprobación de cada una de las actividades curriculares para todos los efectos será 4,0, con aproximación.*

Las calificaciones parciales, las de presentación a actividad final y la nota de actividad final se colocarán con centésima. La nota final de la actividad curricular se colocará con un decimal para las notas aprobatorias, en cuyo caso el 0,05 o mayor se aproximará al dígito superior y el menor a 0,05 al dígito inferior.

Artículo 25: *El alumno(a) que falte sin la debida justificación a cualquier actividad evaluada, será calificado automáticamente con la nota mínima de la escala (1,0).*

Artículo 26: *La calificación de la actividad curricular se hará sobre la base de los logros que evidencie el estudiante en las competencias establecidas en ellos. La calificación final de los diversos cursos y actividades curriculares se obtendrá a partir de la ponderación de las calificaciones de cada unidad de aprendizaje y de la actividad final del curso si la hubiera.*

La nota de aprobación mínima es de 4,0 y cada programa de curso deberá explicitar los requisitos y condiciones de aprobación previa aceptación del Consejo de Escuela.

Artículo 29: *Aquellos cursos que contemplan una actividad de evaluación final**, el programa deberá establecer claramente las condiciones de presentación a esta.***

1. *Será de carácter obligatoria y reprobatoria.*
2. *Si la nota es igual o mayor a 4.0 el estudiante tendrá derecho a dos oportunidades de evaluación final.*
3. *Si la nota de presentación a evaluación final está entre 3.50 y 3.94 (ambas incluidas), el estudiante sólo tendrá una oportunidad de evaluación final.*
4. *Si la nota de presentación es igual o inferior a 3.44**,** el estudiante pierde el derecho a evaluación final, reprobando el curso. En este caso la calificación final del curso será igual a la nota de presentación.*
5. *Para eximirse de la evaluación final, la nota de presentación no debe ser inferior a 5,0 y debe estar especificado en el programa cuando exista la eximición del curso.*

*Según lo dispuesto en el artículo anterior, para este curso, **especificar requisitos particulares para este curso***

El desarrollo de los Informes y trabajos asignados en cada uno de los Módulos deberán ser desarrollados por todos los integrantes del equipo MIIM para poder ser evaluados.

Reglamento general de los planes de formación conducentes a las Licenciaturas y títulos profesionales otorgados por la Facultad de Medicina, D.U. Nº003625 de 27 de enero de 2009.

REGLAMENTO DE ASISTENCIA

Actividades obligatorias

Las inasistencias debidamente justificadas a estas actividades deberán recuperarse de acuerdo con la disponibilidad de tiempo, docentes y campo clínico. Si ellas, por su naturaleza o cuantía, son irrecuperables, el alumno debe cursar la asignatura en su totalidad en el próximo período académico, en calidad de Pendiente o Reprobado, según corresponda.

a) *El estudiante que sobrepase el máximo de inasistencias permitido, figurará como "Pendiente" en el Acta de Calificación Final de la asignatura, siempre que a juicio del PEC, o el Consejo de Nivel o el Consejo de Escuela, las inasistencias con el debido fundamento, tengan causa justificada (Ej, certificado médico comprobable, informe de SEMDA, causas de tipo social o familiar acreditadas por el Servicio de Bienestar Estudiantil.*
b) *El estudiante que sobrepase el máximo de inasistencias permitido, y no aportó elementos de juicio razonables y suficientes que justificaran el volumen de inasistencias, figurará como "Reprobado" en el Acta de Calificación Final de la Asignatura con nota final 3.4.*

Evaluaciones

La inasistencia a una evaluación deberá ser comunicada por la vía más expedita (telefónica – electrónica) en un plazo máximo de 24 horas, posterior a la fecha de la actividad programada.

La justificación de las inasistencias deberá ser presentada en la Secretaría de la Escuela dentro del plazo de 5 días hábiles, contados desde la fecha de la inasistencia, certificada por los Servicios autorizados de la Facultad: Servicio Médico y Dental de los Alumnos; Servicio de Bienestar Estudiantil y Dirección de la Escuela.

Si la justificación se realiza en los plazos estipulados y su PEC acoge la justificación, la actividad deberá ser recuperada preferentemente en forma oral frente a comisión y de carácter acumulativo.
Si no se realiza esta justificación en los plazos estipulados, el estudiante debe ser calificado con la nota mínima (1,0) en esa actividad de evaluación.
Reglamento General de Estudios de las Carreras de la Facultad de Medicina, D.E. Nº 0010109 de 27 agosto de 1997.

Resolución N°1466 "Norma operativa sobre inasistencia a actividades curriculares obligatorias para los estudiantes de pregrado de las Carreras de la Facultad de Medicina. 16 de octubre de 2008.

POLÍTICA DE CORRESPONSABILIDAD SOCIAL EN LA CONCILIACIÓN DE LAS RESPONSABILIDADES FAMILIARES Y LAS ACTIVIDADES UNIVERSITARIAS

Con el fin de cumplir con los objetivos de Propender a la superación de las barreras culturales e institucionales que impiden un pleno despliegue, en igualdad de condiciones, de las mujeres y hombres en la Universidad y el país; Garantizar igualdad de oportunidades para la participación equitativa de hombres y mujeres en distintos ámbitos del quehacer universitario; Desarrollar medidas y acciones que favorezcan la corresponsabilidad social en el cuidado de niñas y niños y permitan conciliar la vida laboral, estudiantil y familiar; y, Desarrollar un marco normativo pertinente a través del estudio y análisis de la normativa universitaria vigente y su eventual modificación, así como de la creación de una nueva reglamentación y de normas generales relativas a las políticas y planes de desarrollo de la Universidad; se contempla cinco líneas de acción complementarias:

Línea de Acción N°1: proveer servicios de cuidado y educación inicial a hijos(as) de estudiantes, académicas(os) y personal de colaboración, facilitando de este modo el ejercicio de sus roles y funciones laborales o de estudio, mediante la instalación de salas cunas y jardines infantiles públicos en los diversos campus universitarios.

Línea de Acción N°2: favorecer la conciliación entre el desempeño de responsabilidades estudiantiles y familiares, mediante el establecimiento en la normativa universitaria de criterios que permitan a los y las estudiantes obtener la necesaria asistencia de las unidades académicas en el marco de la corresponsabilidad social en el cuidado de niñas y niños.

Línea de Acción N°3: garantizar equidad de género en los procesos de evaluación y calificación académica, a través de la adecuación de la normativa| universitaria respectiva, con el fin de permitir la igualdad de oportunidades entre académicas y académicos en las distintas instancias, considerando los efectos de la maternidad y las responsabilidades familiares en el desempeño y la productividad tanto profesional como académico, según corresponda.

Sesión	Horario	Actividades Principales
**UNIDAD I Introducción a la Educación Interprofesional**		
**Sesión 1: 22 Abril**<br><br>15:00 – 16:30 EQUIPO 1 CON PEC<br><br>16:45 - 18:15 EQUIPO 1<br>TRABAJO CON SU EQUIPO SIN PEC	Actividad Sincrónica<br>Trabajo con grupos pequeños con PEC<br>La tarde se comparte con 2 equipos	**PRESENCIAL EQUIPO 1**<br>Bienvenida al curso y revisión de los puntos centrales del Programa de curso<br>Responder la guía de preguntas con la participación de todos los/las/les integrantes de su equipo de trabajo y subirla al ícono tarea disponible en U cursos.
15:00 – 16:30 EQUIPO 2 TRABAJO CON SU EQUIPO SIN PEC<br><br>16:45 - 18:15 EQUIPO 2 CON PEC	Actividad Sincrónica<br>Trabajo con grupos pequeños con PEC<br>La tarde se comparte con 2 equipos	**PRESENCIAL EQUIPO 2**<br>Bienvenida al curso y revisión de los puntos centrales del Programa de curso<br>Responder la guía de preguntas con la participación de todos los/las/les integrantes de su equipo de trabajo y subirla al ícono tarea disponible en U cursos.
**Sesión 2: 29 Abril**<br><br>15:00 a 18:00 horas<br>EQUIPO 1 Y 2	Los estudiantes se organizan y trabajan de manera sincrónica durante la sesión, ambos equipos trabajarán de manera simultánea durante la tarde en el horario de 15:00 a 18:00 horas	Desarrollar la guía de preguntas<br>Cada estudiante del equipo MIIM deberá traer para la sesión la información sobre el rol profesional de su carrera<br><br>Subir la guía desarrollada por escrito al ícono tareas en U cursos.<br><br>PEC disponible para consultas en caso necesario.<br>Envía link a los estudiantes para sus reuniones
**Sesión 3: 6 Mayo**	Actividad sincrónica primera	**PRESENCIAL EQUIPO 1**

15:00 – 16:30 EQUIPO 1 CON PEC<br><br>16:45 - 18:15 EQUIPO 1<br>TRABAJO CON SU EQUIPO SIN PEC	mitad con el PEC<br>Segunda mitad trabajan junto a su equipo	Presentar el trabajo de los roles realizado en la sesión anterior<br><br>Crear mensajes clave relacionados al Trabajo en equipo interprofesional con fotos o un video que deberá subir a Instagram en el que les comparta información a otros.
15:00 – 16:30 EQUIPO 2 TRABAJO CON SU EQUIPO SIN PEC<br><br>16:45 - 18:15 EQUIPO 2 CON PEC	Actividad sincrónica primera mitad con el PEC<br>Segunda mitad trabajan junto a su equipo	**PRESENCIAL EQUIPO 2**<br>Presentar el trabajo de los roles realizado en la sesión anterior<br>Crear mensajes clave<br><br>relacionados al Trabajo en equipo interprofesional con fotos o un video que deberá subir a Instagram en el que les comparta información a otros
**Sesión 4 : 13 Mayo**	TIEMPO PROTEGIDO DE LOS ESTUDIANTES PARA TRABAJAR EN EL CASO FINAL INTEGRADOR MÓDULO	Los estudiantes se reúnen para trabajar junto a su equipo en la resolución del caso clínico
**Sesión 5: 20 Mayo**<br><br>15:00 – 16:30 EQUIPO 1 CON PEC<br><br>16:45 - 18:15 EQUIPO 1<br>TRABAJO CON SU EQUIPO SIN PEC	Actividad Sincrónica con PEC	**PRESENCIAL EQUIPO 1**<br>Presentación del caso final al PEC , posterior a la sesión subir al ícono tarea de U cursos<br><br>El PEC aplica pautas de evaluación
15:00 – 16:30 EQUIPO 2 TRABAJO CON SU EQUIPO SIN PEC<br><br>16:45 - 18:15 EQUIPO 2 CON PEC	Actividad Sincrónica con PEC	**PRESENCIAL EQUIPO 2**<br>Presentación del caso final al PEC , posterior a la sesión subir al ícono tarea de U cursos<br><br>El PEC aplica pautas de evaluación
**UNIDAD II Habilidades para el trabajo en equipo interprofesional y práctica colaborativa**		

**Sesión 6: 27 Mayo**	Los estudiantes se organizan y desarrollan la actividad de manera sincrónica sin el PEC. PEC disponible para consultas	Los estudiantes desarrollan un mapa conceptual y publicar en instagram
**Sesión 7: 3 Junio**<br>15:00 – 16:30 EQUIPO 1 CON PEC<br><br>16:45 - 18:15 EQUIPO 1<br>TRABAJO CON SU EQUIPO SIN PEC	Los estudiantes trabajar en equipo junto a sus compañeros realizando distribución de tareas de manera equitativa y colaborativa	**PRESENCIAL EQUIPO 1**<br>Observar video y leer artículo Resolver la Pauta guía junto a su equipo durante la sesión sincrónica y PEC<br><br>Dar 30 minutos previos para observar el video antes del inicio de la sesión sincrónica.<br><br>Equipo 1 : 15:30 a 16:30<br>Equipo 2 : 16:30 a 17:30
15:00 – 16:30 EQUIPO 2 TRABAJO CON SU EQUIPO SIN PEC<br><br>16:45 - 18:15 EQUIPO 2 CON PEC	Los estudiantes trabajar en equipo junto a sus compañeros realizando distribución de tareas de manera equitativa y colaborativa	**PRESENCIAL EQUIPO 2**<br>Observar video y leer artículo Resolver la Pauta guía junto a su equipo durante la sesión sincrónica y PEC<br><br>Dar 30 minutos previos para observar el video antes del inicio de la sesión sincrónica.<br><br>Equipo 1 : 15:30 a 16:30<br>Equipo 2 : 16:30 a 17:30
**Sesión 8: 10 Junio**<br>15:00 – 16:30 EQUIPO 1 CON PEC<br><br>16:45 - 18:15 EQUIPO 1<br>TRABAJO CON SU EQUIPO SIN PEC	Trabajo de los estudiantes con PEC en la actividad de la sesión.	**PRESENCIAL EQUIPO 1**<br>Los estudiantes leen artículo y analizan noticia<br>Resolver la Pauta guía junto a su equipo durante la sesión sincrónica y PEC
15:00 – 16:30 EQUIPO 2 TRABAJO CON SU EQUIPO SIN PEC<br><br>16:45 - 18:15 EQUIPO 2 CON PEC	Trabajo de los estudiantes con PEC en la actividad de la sesión.	**PRESENCIAL EQUIPO 2**<br>Los estudiantes leen artículo y analizan noticia<br>Resolver la Pauta guía junto a su equipo durante la sesión sincrónica y PEC
**14 y 18 Junio**<br>**Pausa Académica**	**Pausa Académica**	**Pausa Académica**

**Sesión 9: 24 Junio**<br>15:00 – 16:30 EQUIPO 1 CON PEC<br><br>16:45 - 18:15 EQUIPO 1<br>TRABAJO CON SU EQUIPO SIN PEC	Trabajo de los estudiantes con PEC en la actividad de la sesión	**PRESENCIAL EQUIPO 1**<br>Los/Las/ Les estudiantes Observan video y leer artículo .Resolver la Pauta guía junto a su equipo durante la sesión sincrónica y PEC
15:00 – 16:30 EQUIPO 2 TRABAJO CON SU EQUIPO SIN PEC<br><br>16:45 - 18:15 EQUIPO 2 CON PEC	Trabajo de los estudiantes con PEC en la actividad de la sesión	**PRESENCIAL EQUIPO 2**<br>Los/Las/ Les estudiantes Observan video y leer artículo .Resolver la Pauta guía junto a su equipo durante la sesión sincrónica y PEC
**Sesión 10: 1 Julio**<br>15:00 – 16:30 EQUIPO 1 CON PEC<br><br>16:45 - 18:15 EQUIPO 1<br>TRABAJO CON SU EQUIPO SIN PEC	Trabajo de los estudiantes con PEC en la actividad de la sesión	**PRESENCIAL EQUIPO 1**<br>Entrevista a un profesional de la salud de manera *sincrónica* en el horario de miim,junto a su docente.<br><br>El PEC aplica pautas de evaluación
15:00 – 16:30 EQUIPO 2 TRABAJO CON SU EQUIPO SIN PEC<br><br>16:45 - 18:15 EQUIPO 2 CON PEC	Trabajo de los estudiantes con PEC en la actividad de la sesión	**PRESENCIAL EQUIPO 2**<br>Entrevista a un profesional de la salud de manera *sincrónica* en el horario de miim,junto a su docente.<br><br>El PEC aplica pautas de evaluación
**UNIDAD III Integración del trabajo interprofesional y seguridad en los cuidados de salud**		
**Sesión 11: 8 Julio**	Los estudiantes se organizan y desarrollan la actividad de manera sincrónica sin el PEC. PEC disponible para consultas	Los/Las/ Les estudiantes Observan video y leer artículo .Resolver la Pauta guía junto a su equipo

**Sesión 12: 15 Julio**<br>15:00 – 16:30 EQUIPO 1 CON PEC<br><br>16:45 - 18:15 EQUIPO 1<br>TRABAJO CON SU EQUIPO SIN PEC	Trabajo con grupos pequeños con PEC<br>La tarde se comparte con 2 equipos	**PRESENCIAL EQUIPO 1**<br>Los/Las/ Les estudiantes comparten el trabajo de la sesión 1 junto a sus compañeros/as y profesor/ra
15:00 – 16:30 EQUIPO 2 TRABAJO CON SU EQUIPO SIN PEC<br><br>16:45 - 18:15 EQUIPO 2 CON PEC	Trabajo con grupos pequeños con PEC<br>La tarde se comparte con 2 equipos	**PRESENCIAL EQUIPO 2**<br>Los/Las/ Les estudiantes comparten el trabajo de la sesión 1 junto a sus compañeros/as y profesor/ra
**Sesión 13: 22 Julio**	Los estudiantes se organizan y desarrollan la actividad de manera sincrónica sin el PEC.<br>PEC disponible para consultas	Los/Las/ Les estudiantes prepararan medios audiovisuales junto a su equipo de trabajo
**Sesión 14: 29 Julio**	Los estudiantes se organizan y desarrollan la actividad de manera sincrónica sin el PEC.<br>PEC disponible para consultas	Los/Las/ Les estudiantes prepararan medios audiovisuales junto a su equipo de trabajo
**Sesión 15: 5 Agosto**<br><br>Primer Bloque de 15:00 a 16:00 horas; Presentan 2 equipos MIIM	Evaluación Final del Curso<br><br>2 PECS y 4 Equipos MIIM<br><br>La tarde se comparte con 2 equipos<br>Primer Bloque de 15:00 a 16:00 horas; Presentan 2 equipos MIIM	**PRESENCIAL EQUIPO 1**<br>**Actividad Final**<br>Fundamentar los contenidos incluidos en el video acerca de los fundamento de la EIP y práctica colaborativa para disminuir los eventos adversos y lograr una práctica de atención interprofesional de salud segura para las persona en el marco de la Pandemia por Covid 19<br><br>El PEC aplica pautas de evaluación
Segundo Bloque de 16:00 a 17.:00 horas, Presentan 2 equipos MIIM	Trabajo con grupos pequeños con PEC<br><br>2 PECS y 4 Equipos MIIM<br><br>La tarde se comparte con 2	**PRESENCIAL EQUIPO 2**<br>**Actividad Final**<br><br>Fundamentar los contenidos incluidos en el video acerca de los fundamento de la EIP y

	equipos<br><br>Segundo Bloque de 16:00 a 17.:00 horas, Presentan 2 equipos MIIM	práctica colaborativa para disminuir los eventos adversos y lograr una práctica de atención interprofesional de salud segura para las persona en el marco de la Pandemia por Covid 19<br><br>El PEC aplica pautas de evaluación

Programa Elaborado por Profesoras Asociadas Sandra Oyarzo y Mónica Espinoza 2021
