# Supplementary material for: Interprofessional health education teacher training at the University of Chile
Source: J Educ Eval Health Prof. 2021 Nov 15;18:30. doi: 10.3352/jeehp.2021.18.30 (PMC8666264; doi:10.3352/jeehp.2021.18.30)
Supplement: Supplementary file 2 — Supplement 2. Interprofessional education facilitator’s syllabus module I at the Faculty of Medicine of the University of Chile in 2021 (in Spanish). [file jeehp-18-30-suppl2.pdf]

1 SESIÓN INTRODUCCIÓN A LA EDUCACIÓN INTERPROFESIONAL		
**SYLLABUS 1 SESIÓN JUEVES 22 DE ABRIL 2021**		
**Actividad de Aprendizaje**	**Recursos y responsables**	**Actividades de Evaluación y/o evidencias**
**Actividad Inicio :**<br>● Saludo del PEC a sus estudiantes, bienvenida al curso y revisión de los puntos centrales del Programa de curso<br>● Presentación de cada uno de los integrantes del equipo y PEC.	Trabajo con grupos pequeños con PEC<br>La tarde se comparte con 2 equipos<br><br>Cada PEC deberá enviar la invitación para participar a sus estudiantes, se sugiere que esta sesión sea grabada para que los estudiantes que no pudieron participar por problemas de conexión tengan acceso a la sesión.	Lista de asistencia de los estudiantes con la finalidad de llevar registro en relación a la posibilidad de participar a través de este dispositivo<br>Solicitamos por favor que cada PEC reporte a la coordinación la situación de cada uno de los estudiantes:<br>● N° de estudiantes que asistieron en relación al total<br>● Disponibilidad de conexión a internet y dispositivo computador o teléfono para poder hacer el curso.<br>● Situación particular de algún estudiante que presente alguna dificultad.
**Actividad de Desarrollo:**<br>• El PEC presenta las características de la sesión y realiza dinámica de presentación.	**Trabajo con PEC**<br>Presentación de cada estudiante y profesor, en el que se les pregunte ¿cómo están ellos y sus familias? ¿ Qué dificultades tienen? ¿Qué esperan de este curso? El PEC deberá dar la palabra a cada uno de los estudiantes.PEC conversa con los estudiantes acerca del programa y sistema de evaluación del curso.<br><br>**Trabajo Sin PEC**<br>A continuación el PEC informa a los estudiantes que en la segunda parte de la tarde ellos deberán realizar las siguientes actividades:<br>-Crear un sistema de de comunicación y trabajo con los	**Producto 1:** Lo/las/ les estudiantes responden la guía de preguntas con la participación de todos los miembros de su equipo de trabajo y la suben al ícono tarea disponible en U cursos.

	miembros de su equipo<br><br>-Leer el artículo Práctica colaborativa interprofesional en salud: “Conceptos clave, factores y percepciones de los profesionales” disponible en su aula<br>-Ver los videos que se encuentran disponibles para esta esta sesión.<br>Videos de EIP (conceptos generales)<br>- Primer video: contextualización de la EIP. (5 min) https://www.youtube.com/watch?v=NcAOl_fYP90<br>- Segundo vídeo: Impacto para el paciente de la EIP. (5 min) disponible en U curso https://drive.google.com/drive/u/1/folders/1pkNIGQqEkrh5C_iGVLxA3S2mbOTLRcpE<br>-<br>-Responder la guía de preguntas con la participación de todos los/las/les integrantes de su equipo de trabajo y subirla al ícono tarea disponible en U cursos.<br>Preguntas:<br>1. Refiérase a los factores que condicionan la práctica colaborativa interprofesional de acuerdo a lo señalado en el artículo y cómo esto influye en el trabajo en equipo.<br><br>2. En el marco sanitario actual ¿De qué manera los espacios de práctica colaborativa pueden favorecer el trabajo de los equipos de salud?<br><br>3. ¿Qué estrategias proponen para avanzar en la satisfacción de las necesidades de salud y	

	sociales de las personas a través del trabajo de los equipos interprofesionales?	
**El PEC recuerda a los estudiantes:**	• Recordar a los estudiantes que la sesión número 2 ellos trabajaran de manera independiente, sin el tutor.<br>Las actividades de la siguiente se encuentran disponibles en la pauta guía de trabajo en aula de u cursos	
